# Dietary Fat Interacts with PCBs to Induce Changes in Lipid Metabolism in Mice Deficient in Low-Density Lipoprotein Receptor

**DOI:** 10.1289/ehp.7280

**Published:** 2004-09-23

**Authors:** Bernhard Hennig, Gudrun Reiterer, Michal Toborek, Sergey V. Matveev, Alan Daugherty, Eric Smart, Larry W. Robertson

**Affiliations:** ^1^Molecular and Cell Nutrition Laboratory, College of Agriculture,; ^2^Graduate Center for Nutritional Sciences,; ^3^Department of Surgery,; ^4^Department of Pediatrics, and; ^5^Department of Cardiovascular Medicine, University of Kentucky, Lexington, Kentucky, USA; ^6^Department of Occupational and Environmental Health, College of Public Health, University of Iowa, Iowa City, Iowa, USA

**Keywords:** atherosclerosis, dietary fat, gene expression, lipid metabolism, PCB, polychlorinated biphenyl, vascular endothelial cells

## Abstract

There is evidence that dietary fat can modify the cytotoxicity of polychlorinated biphenyls (PCBs) and that coplanar PCBs can induce inflammatory processes critical in the pathology of vascular diseases. To test the hypothesis that the interaction of PCBs with dietary fat is dependent on the type of fat, low-density lipoprotein receptor–deficient (LDL-R^−/−^) mice were fed diets enriched with either olive oil or corn oil for 4 weeks. Half of the animals from each group were injected with PCB-77. Vascular cell adhesion molecule-1 (VCAM-1) expression in aortic arches was non-detectable in the olive-oil–fed mice but was highly expressed in the presence of PCB-77. PCB treatment increased liver neutral lipids and decreased serum fatty acid levels only in mice fed the corn-oil–enriched diet. PCB treatment increased mRNA expression of genes involved in inflammation, apoptosis, and oxidative stress in all mice. Upon PCB treatment, mice in both olive- and corn-oil–diet groups showed induction of genes involved in fatty acid degradation but with up-regulation of different key enzymes. Genes involved in fatty acid synthesis were reduced only upon PCB treatment in corn-oil–fed mice, whereas lipid transport/export genes were altered in olive-oil–fed mice. These data suggest that dietary fat can modify changes in lipid metabolism induced by PCBs in serum and tissues. These findings have implications for understanding the interactions of nutrients with environmental contaminants on the pathology of inflammatory diseases such as atherosclerosis.

From epidemiologic studies, there is substantial evidence that cardiovascular diseases are linked to environmental pollution and that exposure to polycyclic and/or polyhalogenated aromatic hydrocarbons can lead to human cardiovascular toxicity. For example, one study found a significant increase in mortality from cardiovascular diseases among Swedish capacitor manufacturing workers exposed to polychlorinated biphenyls (PCBs) for at least 5 years (Gustavsson and Hogstedt 1997), and in another study most excess deaths were due to cardiovascular disease in power workers exposed to phenoxy herbicides and PCBs in waste transformer oil (Hay and Tarrel 1997). The increased prevalence of atherosclerosis may be associated with the ability of PCBs to modulate plasma and tissue lipids, events that can result in compromised lipid metabolism and lipid-dependent cellular signaling pathways. In a study with rhesus monkeys, Bell et al. (1994) found a causal relationship between plasma lipid changes and PCB intake after oral exposure of Aroclor 1254. Moreover, a report by Tokunaga et al. (1999) confirms many other studies with chronic Yusho patients (accidental ingestion of rice-bran oil contaminated with PCBs), which showed in this population that elevated serum levels of triglycerides and total cholesterol were significantly associated with the blood PCB levels. Serum lipids also have been shown to be affected by PCBs, which apparently can modify the regulatory mechanisms of synthesis and degradation of cholesterol (Jenke 1985). A major route of exposure to PCBs in humans is via oral ingestion of contaminated food products (Safe 1994). Therefore, circulating environmental contaminants derived from diets, such as PCBs, are in intimate contact with the vascular endothelium.

In addition to serum and vascular lipid changes, a number of studies have reported an increase in liver and hepatic microsomal lipids (total lipids, phospholipids, neutral lipids, and cholesterol) after PCB administration (Garthoff et al. 1977; Ishidate et al. 1978). Asais-Braesco et al. (1990) reported that a single injection of PCB-77 resulted in a marked change in the fatty acid composition of rat hepatic microsomal fractions. Also, Matsusue et al. (1999) found that coplanar PCBs have a significant effect on the reduced synthesis of physiologically essential long-chain unsaturated fatty acids, such as arachidonic acid in rat liver, by suppressing delta-5 and delta-6 desaturase activities and thus allowing the omega-6 parent fatty acid, linoleic acid, to accumulate.

Little is known about the interaction of dietary fats and PCBs in the pathology of atherosclerosis. We have reported a significant disruption in endothelial barrier function when cells were exposed to linoleic acid (Hennig et al. 2001a). In addition to endothelial barrier dysfunction, another functional change in atherosclerosis is the activation of the endothelium that manifests as an increase in the expression of specific cytokines and adhesion molecules. These cytokines and adhesion molecules are proposed to mediate the inflammatory aspects of atherosclerosis by regulating the vascular entry of leukocytes. We reported previously that coplanar PCBs and linoleic acid induce the expression of cytokines and adhesion molecules in cultured endothelial cells (Hennig et al. 2002; Toborek et al. 2002). In addition, both linoleic acid and PCB-77—and more markedly when applied in combination—can generate reactive oxidative species that can trigger oxidative-stress–sensitive proinflammatory signaling pathways (Hennig et al. 2002a). These studies suggest that environmental contaminants such as PCBs are atherogenic in part by their ability to alter endothelial cell lipid profile and metabolism and by inducing oxidative stress and pro-inflammatory genes.

Exposure to physiologic concentrations of specific fatty acids, such as linoleic acid, can trigger inflammatory pathways leading to the up-regulation of inflammatory cytokines [e.g., interleukin-6 (IL-6), IL-8] and adhesion molecules [e.g., vascular cell adhesion molecule-1 (VCAM-1), E-selectin]. These genes initiate the chemoattraction and adhering of monocytes, events occurring early in the pathogenesis of atherosclerosis. The differential effect of various fatty acids is most likely due to different susceptibility to oxidation and thus generation of oxidative stress as well as their role in precursors of lipid-derived second messengers (Hennig and Toborek 2001). Therefore, we hypothesize that selected dietary lipids may modulate the atherogenicity of environmental chemicals by interfering with metabolizing and inflammatory pathways and thus leading to dysfunction of the vasculature and related tissues.

The present data indicate that dietary fat can modify changes in lipid metabolism induced by PCB in a low-density-lipoprotein (LDL)-receptor–deficient (LDL-R^−/−^) mouse model, that is, mice that develop atherosclerosis as a result of increased sensitivity to different types of dietary fat (Daugherty 2002). Our data also support our hypothesis that dietary oils rich in linoleic acid can further compromise gene expression during PCB cytotoxicity.

## Materials and Methods

### Animal model and PCB treatment.

The LDL-R^−/−^ mice used in this study were originally obtained from the Jackson Laboratory (stock no. 002207; Bar Harbor, ME) and bred at the University of Kentucky. LDL-R^−/−^mice have become a preferred model for atherosclerosis because their elevated LDL fraction resembles the lipoprotein profile of hypercholesterolemic humans (Daugherty 2002). All animal procedures were in compliance with the institutional animal care and use committee guidelines of the University of Kentucky. Mice were divided into four groups of five mice per treatment: olive-oil–rich diet, olive-oil–rich diet plus PCB injection, corn-oil–rich diet, and corn-oil–rich diet plus PCB injection. Mice were injected intraperitoneally with PCB-77 [170 μmol/kg body weight (bw)] or the vehicle (olive oil or corn oil; Dyets Inc., Bethlehem, PA) at weeks 1 and 3 of the 4-week feeding study.

After completion of the study, animals were euthanized using intraperitoneal ketamine injections. Serum and aortic and liver tissues were obtained for analysis. According to our combined experience with several animal species, long-term intraperitoneal injections of 100–300 μmol/kg bw per injection are sufficient to initiate disease states, such as tumor promotion (Robertson et al. 1991). In our preliminary studies, we saw adhesion molecule expression at 170 μmol/kg bw per injection; thus, this concentration was chosen for the present study. This amount of PCB was based on calculated values from our *in vitro* experiments that were themselves based on levels that are usually found in humans after acute exposure (Jensen 1989; Wassermann et al. 1979).

### Experimental diets.

We chose corn and olive oils because of previous cell culture work with individual fatty acids (Toborek et al. 2002). In these experiments, linoleic acid was able to amplify the inflammatory response of endothelial cells exposed to PCB-77. In addition, we have evidence that a high-corn-oil diet is proinflammatory and induces atherosclerotic pathology relative to a high-olive-oil diet (B. Hennig, unpublished data). We therefore chose corn oil because it contains about 50% linoleic acid as triglycerides, and thus is a significant dietary source of linoleic acid. As a control, we chose olive oil, with the predominant fatty acid being oleic acid. Oleic acid is also an 18-carbon fatty acid but acted “neutral” when endothelial cells were coexposed to oleic acid and PCB-77 (B. Hennig, unpublished data). In fact, our previous studies suggest that oleic acid has little effect or even can decrease an inflammatory response (Toborek et al. 2002).

**Figure 1:**
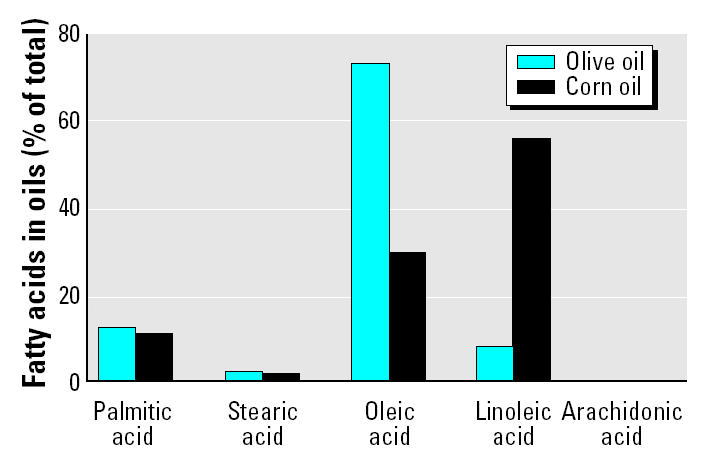
Fatty acid analysis of the two oils used in the feeding study. Fatty acids are measured in g/100 g total fatty acids; palmitic acid, 16:0; stearic acid, 18:0; oleic acid, 18:1; linoleic acid, 18:2; arachidonic acid, 20:4.

Diets were custom prepared and vacuum packed (Dyets Inc.). Diets were based on a modified AIN-76A purified rodent diet (Reeves 1997) with varying sources of fat. The dietary fat content, either olive oil or corn oil, was 150 g/kg total diet. The antioxidant content of each oil was adjusted by the manufacturer. The fatty acid composition in the different oils is shown in Figure 1.

### Serum fatty acid analysis.

Total plasma lipids were extracted by the method of Bligh and Dyer (1959) as modified by Williams et al. (1984). Internal standard (heneicosanoic acid, 5 μg in methanol) was added to the samples before lipid extraction. All solvents for liquid extraction contained 50 mg/L butylated hydroxytoluene as an antioxidant (Silversand and Haux 1997). Lipids were dried under nitrogen followed by fatty acid esterification with boron trifluoride–methanol. Fatty acid methyl esters were extracted with hexane for gas chromatography injection. The gas chromatograph (Agilent 6890 GC G2579A system; Agilent Technologies, Palo Alto, CA) was equipped with an OMEGAWAX 250 capillary column. The following temperature program was used: 160°C for 5 min, an increase in temperature to 220°C at a rate of 2°C/min, followed by 220°C for 15 min. A model 5973 mass-selective detector (Agilent Technologies) was used for detection of separated lipids.

### Neutral lipid staining of liver tissues.

Liver sections were fixed overnight in 4% para-formaldehyde in phosphate-buffered saline (PBS) before embedding in OCT (optimal cutting temperature) compound (Fisher Scientific, Pittsburgh, PA). Serial (10 μm) sections were mounted on MicroProbe slides (Fisher Scientific), and neutral lipids were stained with Oil Red O, as described previously (Daugherty et al. 2000).

### Immunostaining of aortic tissue.

Aortic tissue from the thoracic regions was excised, immersed in OCT embedding medium, and frozen at −20°C, and 8 μm sections were cut on a cryostat. Immunocytochemistry was performed as described previously (Daugherty et al. 2000). Briefly, endogenous peroxidase was inactivated using hydrogen peroxide (3%) in methanol. Samples were blocked in the serum of the secondary antibody host. Primary antibodies for VCAM-1 (PharMingen, San Diego, CA) were detected using biotinylated secondary antibodies and peroxidase ABC kits (Vectastain, Burlingame, CA). Aminoethylcarbazole was used as chromogen, and sections were counterstained with hematoxylin.

### Gene expression analysis.

For microarray analysis, total RNA was isolated from snap frozen liver tissue using RNAeasy (Quiagen, Valencia, CA). RNA samples were pooled for analysis of two data sets per treatment group. RNA integrity analysis and biotin-labeling of cRNA was performed by the Microarray Core Facility at the University of Kentucky. Labeled RNA was spotted on Murine Genome MOE 430 chips and detected in the Affymetrix 428 fluorescence reader (both from Affymetrix, Santa Clara, CA).

Microarray data were confirmed by conventional reverse-transcription polymerase chain reaction (RT-PCR). RNA was isolated from liver samples. cDNA was generated by RT and amplified by PCR using the following primers: cytochrome P450 1A1 (CYP1A1), forward 5′-CAGATGATAAGGTCAT-CACGA-3′, reverse 5′-TTGGGGATAT-AGAAGCCATTC-3′; acetyl-coenzyme A (CoA)-carboxylase, forward 5′-ACAG-TGAAGGCTTACGTCTG-3′, reverse 5′-AGGATCCTTACAACCTCTGC-3′; and β-actin, forward 5′-ATGGATGAC-GATATCGCT-3′, reverse 5′-ATGAGG-TAGTCTGTCAGGT-3′. PCR products were separated on a 2% agarose gel, stained with SYBR gold, and visualized using a phosphoimager (Fuji FLA-5000; Fuji Medical Systems, Stamford, CT).

### Quantitations and statistical analyses.

Numeric data were analyzed using SYSTAT 7.0 (SPSS, Inc., Chicago, IL). Comparisons between treatments were made by one-way ANOVA with post hoc comparisons of the means made by Bonferroni least significance difference procedure. Student *t*-tests were employed to compare gene expression data showing a PCB-dependent change. Statistical probability of *p* < 0.05 was considered significant.

Photomicrographs of VCAM-1 and neutral lipid staining in aortic roots and livers, respectively, were evaluated by individuals who were blinded to the specimen identification.

## Results

### PCB treatment increases diet-dependent clearance of serum fatty acids.

**Figure 2:**
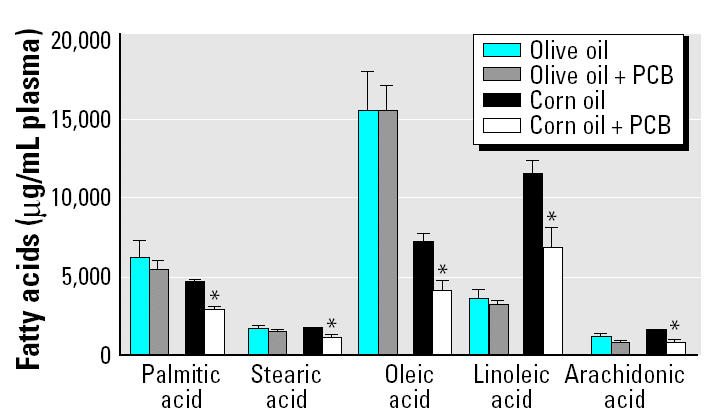
Fatty acid profile in serum. See “Materials and Methods” for details. Values are mean ± SEM (*n* = 5). Palmitic acid, 16:0; stearic acid, 18:0; oleic acid, 18:1; linoleic acid, 18:2; arachidonic acid, 20:4.
*Significantly different from respective diet treatment without PCBs.

As expected, feeding a diet enriched with olive oil or corn oil resulted in serum fatty acid profiles (Figure 2) comparable with the fatty acid profile in the respective oils (Figure 1). PCB treatment had little effect on fatty acid patterns in animals fed the olive oil diet. In contrast, PCB treatment of corn-oil–fed mice resulted in marked decreases in major serum fatty acids, with a quantitatively most significant serum clearance of serum linoleic acid.

### PCBs increase neutral lipid staining in liver tissue.

**Figure 3:**
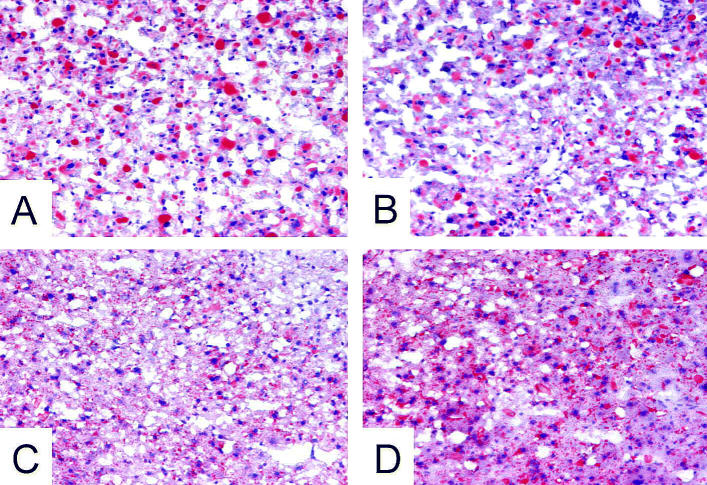
Lipid staining of mouse liver sections. (*A*) Olive oil. (*B*) Olive oil plus PCB. (*C*) Corn oil. (*D*) Corn oil plus PCB. See “Materials and Methods” for details. Magnification, 200×.

Baseline or control lipid staining (Oil Red O) appeared to be similar in liver tissues from both olive-oil– and corn-oil–fed mice. In contrast to the olive oil group, PCB exposure further increased neutral lipid staining only in LDL-R^−/−^ mice fed the corn-oil–enriched diet (Figure 3).

### VCAM-1 expression is affected by diet and PCBs.

**Figure 4:**
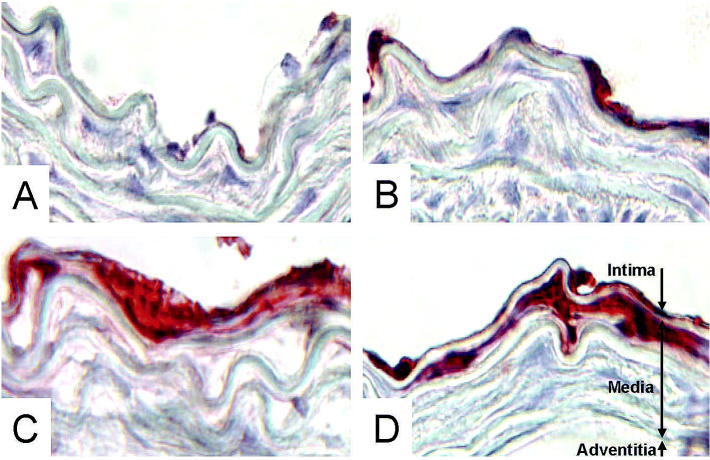
Immunoreactivity of VCAM-1 antiserum against sections of mouse aortic arches. (*A*) Olive oil. (*B*) Olive oil plus PCB. (*C*) Corn oil. (*D*) Corn oil plus PCB. See “Materials and Methods” for details. Red staining reflects positive chromogen development for VCAM-1 immunostaining on the endothelial surface (*B*–*D*) and in subendothelial tissue (*D*). Magnification, 400×.

VCAM-1 expression was negligible in mice fed the olive-oil–enriched diet (Figure 4A), whereas, corn-oil–fed mice exhibited elevated VCAM-1 expression (Figure 4C). In corn-oil–fed mice, PCB treatment further increased VCAM-1 staining in aortic tissues (Figure 4D). PCB treatment markedly increased VCAM-1 expression at the vascular surface in all animals, independent of dietary fat. Interestingly, PCB treatment increased VCAM-1 expression in smooth-muscle–rich areas of the vessel in mice fed the corn-oil–enriched diet (Figure 4D). This phenomenon was not observed in mice fed the olive-oil–enriched diet.

### Gene expression change in response to PCB-77 in mice fed a high-corn or high-olive-oil diet.

PCB treatment markedly increased expression of selected genes involved in inflammation, apoptosis, and oxidative stress in both diet groups (Table 1). Data represent expression values of both dietary groups compared with both dietary groups receiving PCBs. The oil-dependent effect of PCB-77 was most apparent in mRNA levels of genes involved in lipid metabolism (Table 2). Feeding diets rich in either corn or olive oil induced fatty acid degradation but with up-regulation of different key enzymes. For example, PCB treatment induced carnitine palmitoyltransferase in corn-oil–fed animals, whereas glycerol-3-P-dehydrogenase and fatty acid CoA ligase 4 were induced in olive-oil–fed mice. Genes involved in fatty acid synthesis, such as acetyl-CoA-carboxylase and elongation of long-chain fatty acids were reduced only by PCB-77 in corn-oil–fed mice, whereas lipid transport/export genes such as fatty acid binding protein 2 and 4, ATP-binding cassette A1, and apolipoprotein A-IV were altered in olive-oil–fed mice in response to PCBs.

**Figure 5:**
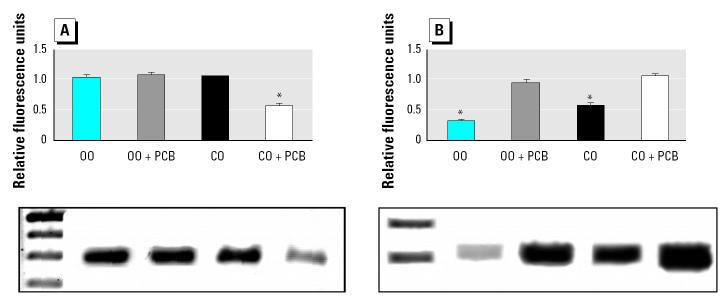
mRNA expression of acetyl-CoA-carboxylase (*A*) and *CYP1A1* (*B*) as analyzed by RT-PCR; gels below show one representative sample per treatment group of RT-PCR. Abbreviations: CO, corn oil; OO, olive oil. See “Materials and Methods” for details. Values are mean ± SEM (*n* = 5); values are normalized to β-actin.
*Significantly different from all other groups, *p* < 0.05.

Microarray analysis of selected genes was confirmed by conventional RT-PCR. For example, PCB treatment only decreased expression of the acetyl-CoA-carboxylase gene in mice fed the corn oil diet (Figure 5A). As expected, PCB treatment increased *CYP1A1* gene expression in all mice (Figure 5B).

## Discussion

There is substantial evidence that environmental pollution can be correlated with the incidence of cardiovascular diseases (Hennig et al. 2001b). This might be due to a PCB-mediated impairment of lipid metabolism. In the vasculature, alterations in lipid profile and lipid metabolism as a result of exposure to PCBs may be important components of endothelial cell dysfunction (Hennig et al. 2002a). Endothelial cell dysfunction is an important factor in the overall regulation of vascular lesion pathology. We have reported recently that PCB-77 can increase expression of cytokines, such as IL-6, and adhesion molecules, such as VCAM-1, in cultured endothelial cells (Hennig et al. 2002b). Little is known about the interaction of dietary fats and PCBs in the pathology of atherosclerosis. We hypothesize that selected dietary lipids, and especially oils rich in linoleic acid, may increase the atherogenicity of environmental chemicals, such as PCBs, by cross-amplifying mechanisms leading to dysfunction of the vasculature and related tissues. Indeed, immunohistochemistry data from the present study demonstrate the cumulative effect of corn oil and PCB-77 on aortic VCAM-1 expression. Although olive-oil–fed mice did not show expression of this adhesion molecule unless they were injected with PCBs, corn oil feeding alone already resulted in a strong staining for VCAM-1. In corn-oil–fed mice injected with PCBs, VCAM-1 expression could even be detected in the sub-endothelial space, suggesting a progressed state of atherosclerosis with adhesion molecule expression on smooth muscle cells. These data are in agreement with epidemiologic studies that suggest diets high in olive oil or oleic acid protect against cardiovascular diseases (Massaro and De Caterina 2002). However, the interaction of different dietary fats with environmental contaminants and the effect on the pathogenesis of atherosclerosis is unknown and has not been studied in LDL-R^−/−^ mice.

There is considerable evidence that exposure to PCBs can lead to lipid changes in plasma and tissues and that this may be linked to lipophylic properties of PCBs and their interaction with lipids and especially with fatty acids. For example, exposure to Aroclor 1242 modified adipose tissue fatty acids, with a decrease of highly unsaturated fatty acids and an increase in monounsaturated fatty acids in membrane phospholipids (Kakela and Hyvarinen 1999). Our microarray analysis of liver mRNA suggests that PCB–lipid interactions are dependent on the type of dietary fat. For example, the PCB-mediated up-regulation of genes involved in fatty acid uptake and catabolism, as well as down-regulation of genes involved in fatty acid synthesis, involved different key enzymes depending on the oil that was used in the diet. It appears that PCBs had more effect on fatty acid synthesis in corn-oil–fed animals, whereas there was a greater change in genes involved in fatty acid transport in olive-oil–fed mice.

Overall, lipid metabolism was affected to a greater extent in corn-oil–fed animals as demonstrated also by serum and liver lipid analyses. Lipids appear to be removed from the plasma and accumulate in tissues in corn-oil–fed animals receiving PCB injection. A number of studies have reported an increase in liver and hepatic microsomal lipids (total lipids, phospholipids, neutral lipids, and cholesterol) after PCB administration (Asais-Braesco et al. 1990; Garthoff et al. 1977; Hinton et al. 1978; Ishidate et al. 1978; Robertson et al. 1991). The amplified toxicity of linoleic acid and PCBs to endothelial cells could thus be mediated by cellular accumulation of this fatty acid and its subsequent transformation to toxic cytotoxic epoxide metabolites (Viswanathan et al. 2003). Because of the very low basal activity of endothelial cell delta-6 desaturase, arachidonic acid is not produced from linoleic acid significantly in this type of cell (Debry and Pelletier 1991; Spector et al. 1981), which can result in linoleic acid accumulation within endothelial cells (Hennig and Watkins 1989; Spector et al. 1981). Furthermore, Matsusue et al. (1999) demonstrated that coplanar PCBs can suppress delta-5 and delta-6 desaturase activities. The decreased expression of the long-chain fatty acid elongase detected in corn-oil–fed mice treated with PCBs also suggests an impairment in fatty acid metabolism. Using endothelial cell culture models, we showed previously that linoleic acid uptake and cellular accumulation of this fatty acid are markedly increased in the presence of PCB-77, further supporting our hypothesis that PCB-induced endothelial cell dysfunction can be modulated by the cellular lipid milieu (Slim et al. 2001).

In summary, our data clearly demonstrate a selective interaction of diet, and especially dietary fats, with PCB-induced cellular functions. These findings may contribute to a better understanding of the interactive mechanisms of dietary fats and environmental contaminants as mediators of vascular endothelial cell dysfunction and vascular pathologies such as atherosclerosis.

## Figures and Tables

**Table 1:** PCB-mediated up-regulation of mRNA expression of selected genes involved in inflammation, apoptosis, and oxidative stress.

	High-fat diets (mean ± SEM)	High-fat diets + PCB (mean ± SEM)	*p*-Value
Inflammation			
Neuronal pentraxin	33.0 ± 5.3	118.2 ± 8.2	0.01
Amyloid beta (A4) precurser	1315.6 ± 3.0	1954.6 ± 2.0	0.12
IL-6 signal transducer	129.6 ± 31.3	229.4 ± 20.5	0.04
IL-2 receptor, gamma chain	177.9 ± 28.6	331.1 ± 42.5	0.02
Matrix metalloproteinase 19	95.8 ± 23.9	124.95 ± 28.9	0.47
Membrane metalloendopeptidase	110.9 ± 13.2	159.13 ± 30.0	0.19
Apoptosis			
Caspase 6	490.9 ± 67.7	703.5 ± 41.6	0.04
Caspase 7	183.8 ± 40.6	326.9 ± 1.9	0.01
Caspase 8 and FADD-like	79.4 ± 13.0	134.0 ± 32.5	0.17
Apoptosis inhibitor 5	83.7 ± 11.5	147.9 ± 13.6	0.01
Oxidative stress			
CYP1A1	692.1 ± 465.0	2999.8 ± 691.1	0.03
CYP1A2	8823.9 ± 2118.8	16927.4 ± 979.0	0.01
NADPH oxidase 4	625.8 ± 150.4	844.5 ± 50.6	0.22
Superoxide dismutase 2	125.1 ± 15.6	204.9 ± 18.3	0.02

**Table 2:** Relative expression changes of genes involved in lipid metabolism upon PCB-77.

Gene	Function	Olive oil	Corn oil
Carnitine palmitoyl-transferase 1	Fatty acid degradation	—	↑↑
Glycerol-3-P-dehydrogenase	Fatty acid degradation	↑↑	—
Fatty acid CoA ligase 4	Fatty acid degradation	↑↑	—
Acetyl-CoA-carboxylase β	Fatty acid synthesis	—	↓↓
Long-chain fatty acyl elongase	Fatty acid elongation	—	↓↓
CD 36	Fatty acid uptake	—	↑
Fatty acid binding protein 4	Fatty acid transport	↓	—
Fatty acid binding protein 2	Fatty acid transport	↓↓	—
ATP-binding cassette A1	Cholesterol export	↓↓	—
Apolipoprotein A-IV	Lipoprotein metabolism	↑↑	—
HDL binding protein	Lipoprotein metabolism	↓↓	—
CYP1A1	Fatty acid metabolism	↑↑	↑↑

Data shown refer to ratios of diet alone compared with diet plus PCB-77 within each dietary treatment: —, no change; ↑ and ↓, ≥1.5-fold change; ↑ ↑ and↓↓, ≥2-fold change.
